# Poly(ethylene glycol)-*b*-poly(1,3-trimethylene carbonate) Copolymers for the Formulation of In Situ Forming Depot Long-Acting Injectables

**DOI:** 10.3390/pharmaceutics13050605

**Published:** 2021-04-22

**Authors:** Marie-Emérentienne Cagnon, Silvio Curia, Juliette Serindoux, Jean-Manuel Cros, Feifei Ng, Adolfo Lopez-Noriega

**Affiliations:** MedinCell SA, 3 Rue des Frères Lumière, 34830 Jacou, France; marie.cagnon@medincell.com (M.-E.C.); silvio.curia@medincell.com (S.C.); juliette.serindoux@medincell.com (J.S.); jeanmanuel.cros@medincell.com (J.-M.C.); feifei.ng@medincell.com (F.N.)

**Keywords:** polycarbonate, long-acting injectable, in situ forming depot, meloxicam

## Abstract

This article describes the utilization of (methoxy)poly(ethylene glycol)-*b*-poly(1,3-trimethylene carbonate) ((m)PEG–PTMC) diblock and triblock copolymers for the formulation of in situ forming depot long-acting injectables by solvent exchange. The results shown in this manuscript demonstrate that it is possible to achieve long-term drug deliveries from suspension formulations prepared with these copolymers, with release durations up to several months in vitro. The utilization of copolymers with different PEG and PTMC molecular weights affords to modulate the release profile and duration. A pharmacokinetic study in rats with meloxicam confirmed the feasibility of achieving at least 28 days of sustained delivery by using this technology while showing good local tolerability in the subcutaneous environment. The characterization of the depots at the end of the in vivo study suggests that the rapid phase exchange upon administration and the surface erosion of the resulting depots are driving the delivery kinetics from suspension formulations. Due to the widely accepted utilization of meloxicam as an analgesic drug for animal care, the results shown in this article are of special interest for the development of veterinary products aiming at a very long-term sustained delivery of this therapeutic molecule.

## 1. Introduction

In situ forming depot (ISFD) long-acting injectables (LAI) have been widely investigated in the last decades as pharmaceutical forms for the sustained delivery of small molecules and biologics [1,2,3,4]. ISFD technologies are based on the transition of a drug product from a liquid to a solid form upon injection into the organism. Different mechanisms might be driving this transition, such as the thermogelling behavior of the excipients of the formulation at body temperature, the physical crosslinking among polymeric chains, or the solvent exchange in between the injectable and the surrounding aqueous medium, to name a few [5,6]. In all cases, the result is the generation of a drug reservoir that will release its cargo by diffusion and/or its degradation. In most cases, ISFD LAIs are designed using bioresorbable materials, which makes them ideal products to ensure long-term drug compliance by the patients while guaranteeing also an increased comfort. It is not surprising thus that several ISFD-based drug products have reached clinics for different indications and that many more are currently undergoing clinical trials [7].

Different materials have been proposed as functional excipients for the formulation of ISFD; among them, polycarbonates have been evaluated in the past. On top of their biocompatibility and bioresorbability, there are other additional features that make these polymers especially attractive for this application. On the one hand, polycarbonates are not hydrolyzed at physiological parenteral conditions but rather undergo enzymatic degradation yielding nonacidic byproducts [8,9]. This is a remarkable characteristic for the long-term delivery of labile molecules such as proteins that might be denatured within the reservoir upon polymeric degradation and subsequent accumulation of acidic degradants [10,11,12]. Indeed, this is a drawback that has been widely described for polyester-based drug delivery technologies, which are degraded by hydrolysis [13]. On the other hand, different studies have demonstrated that polycarbonate-based materials are degraded by surface erosion [14]. Thus, it can be expected that, in the case of ISFD, only the drug molecules within the depot that are gradually becoming closer to the outer surface of the polymeric reservoirs upon degradation will be delivered by diffusion into the surrounding medium. This is a major difference from depots that undergo bulk erosion, on which drug diffusion is likely to happen through the full volume of the materials [15]. This feature makes these polycarbonates singularly interesting for the release of drugs with high aqueous solubility.

Based on the above, polycarbonate homopolymers such as poly(1,3-trimethylene carbonate) (PTMC) or poly(ethylene carbonate) (PEC) have been tested for the formulation of ISFD LAI by solvent exchange, demonstrating their potential as drug delivery technologies [16,17,18]. As a matter of fact, several studies evaluated the biocompatibility and bioresorption of thus formed drug reservoirs, confirming their usefulness for the preparation of LAIs. However, by using polycarbonate homopolymers, the number of parameters in the formulation that may be tuned to control the release kinetics and profile is very limited (i.e., polymer molecular weight and polymer content within the formulation) [19]. One way to overcome this limitation would be the utilization of polycarbonate-based copolymers with a hydrophilic block such as poly(ethylene glycol) (PEG). Similar to other copolymers such as PEG-polyesters, formulating with amphiphilic PEG–polycarbonate block copolymers would yield increased flexibility to ultimately achieve a wider range of delivery duration and kinetics; the hydrophilic:hydrophobic ratio within the copolymers would be an additional parameter to leverage in order to further adjust the final functionality of the drug product [20,21].

This article describes the utilization of (methoxy)poly(ethylene glycol)-*b*-poly(1,3-trimethylene carbonate) ((m)PEG–PTMC) diblock (DB) and triblock (TB) copolymers for the formulation of ISFD LAIs by solvent exchange. Two active pharmaceutical ingredients (API), tamsulosin and meloxicam, were used as model therapeutic molecules to evaluate the adequacy of these formulations to afford a sustained delivery in vitro and assess the feasibility of modulating the release kinetics by using different (m)PEG–PTMC copolymers. Selected (m)PEG–PTMC-based LAI formulations with meloxicam were then tested in a 28-day pharmacokinetic study in rats to assess the long-term delivery of this drug. The internal structure of the depots and the polymer integrity upon in vivo administration were analyzed with the aim of giving an insight into the phenomena driving the drug release and the degradation of these materials. Due to the widely accepted utilization of meloxicam as an analgesic drug for animal care, the results shown in this article are of special interest for the development of veterinary products aiming at a very long-term sustained delivery of this therapeutic molecule [22,23,24].

## 2. Materials and Methods

### 2.1. Materials and Nomenclature

**Figure 1:**
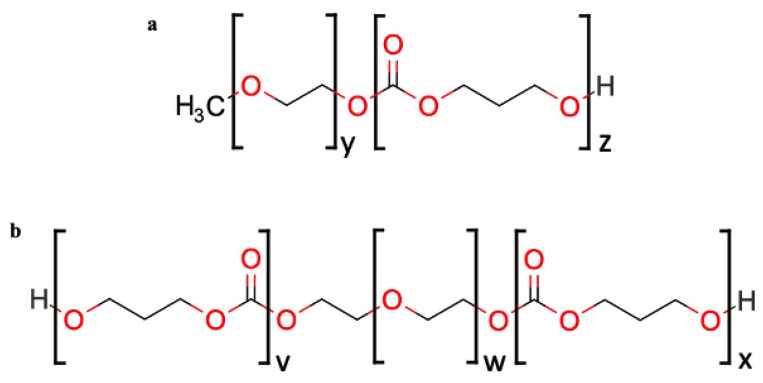
Chemical structure of a diblock (**a**) and a triblock (**b**) PEG–PTMC copolymer.

DB mPEG–PTMC and TB PTMC–PEG–PTMC copolymers, whose chemical structure is depicted in Figure 1, were synthesized at MedinCell. Details around the synthesis and characterization of such polymers can be found elsewhere [25]. Pharma grade acetonitrile was acquired from Carlo Erba (Peypin, France) and USP grade dimethyl sulfoxide (DMSO) was purchased from Gaylord Chemical (Los Angeles, CA, USA).

Copolymers used in this study were coded as TBm-n and DBs-t, where m and s correspond to the molecular weight, in kDa, of the PEG and mPEG of TB and DB, respectively, and n and t correspond to the molecular weight, in kDa, of the total PTMC within the copolymer. For instance, TB1-9.3 stands for a triblock copolymer with 1 kDa PEG and 9.3 kDa PTMC; DB0.35-6.9 stands for a diblock copolymer with 0.35 kDa mPEG and 6.9 kDa PTMC.

Meloxicam (Mw = 351.41 g/mol) was purchased from Interchim (Montluçon, France) and tamsulosin hydrochloride (Mw = 444.97 g/mol) was purchased from Capot Chemical (Hangzhou, China). Both were reagent grades. The solubility at room temperature of meloxicam and tamsulosin in phosphate-buffered saline (PBS) buffer at pH = 7.4, used during the in vitro release tests, is 400 µg/mL and 2300 µg/mL, respectively. After 3 days under continuous agitation at room temperature of saturated solution, the solubility in PBS buffer was determined in-house by measuring the concentration of the drugs in the filtered supernatant. The ultra-performance liquid chromatography (UPLC) methods described in the Supplementary Materials (Tables S1 and S2) were used to perform these analyses.

All chemicals were used as received without further purification. Tamsulosin was cryo-milled before use in order to obtain a powder with a homogeneous particle size distribution (data not shown).

Formulations were coded Fa: z%TBm-n_y%API or Fa: z%DBs-t_y%API, where a is the incremental number relative to the tested formulation; m, n, s, and t correspond to the molecular weights in kDa of (m)PEG and PTMC in the copolymers; z and y depict, respectively, the amount of copolymer and API in the formulation as % mass of the total formulation mass (i.e., polymer + API + solvent).

For example, F05: 40.0%TB1-9.3_8.0% meloxicam means that the formulation 05 was produced with a triblock with 1 kDa PEG and 9.3 kDa PTMC; the weight fraction of copolymers and API in the formulation are, respectively, 40.0% and 8.0% (wt%).

Table 1 and Table 2 summarize the composition of meloxicam and tamsulosin formulations, respectively, tested in this study.

All tests described in this section were carried out in triplicate (n = 3) unless otherwise indicated.

All results are shown as the average ± standard deviation unless otherwise indicated.

### 2.2. Meloxicam and Tamsulosin In Vitro Release Tests

In vitro release kinetics from the resulting depots of two model molecules—meloxicam and tamsulosin—were assessed to determine the effect of using different compositions in the formulations.

A volume of 50 µL of the formulation was injected using a syringe without needle into a 50 mL prefilled glass vial containing 40 mL or 20 mL of PBS release buffer at pH = 7.4 for meloxicam and tamsulosin, respectively. Once the polymer precipitation and depot formation had occurred upon contact with the aqueous buffer (ca. 2 min), the depot was cut from the syringe using a pair of scissors. The closed glass vials were kept at 37 °C under continuous orbital shaking.

At predetermined time points, 2 mL of the release medium were withdrawn and kept for further analysis. At each sampling, all the release medium was renewed with fresh buffer. Samples were kept at 4 °C until analysis. Sink conditions were maintained through the full duration of the study (i.e., concentration in the medium was always lower than 1/10th of the saturation concentration of the drugs).

Reverse-phase (RP)–UPLC was used to determine the concentration of meloxicam or tamsulosin in the release medium following the methods summarized in the Supplementary Materials (Tables S1 and S2). Prior to analysis, the different samples were filtered through a 0.2 µm hydrophilic filter.

At the end of the in vitro release tests, depots were recovered from the medium and dissolved for one hour in a mix of acetonitrile and water (4:1 ratio for meloxicam and 3:7 for tamsulosin; *v*:*v*), in order to determine the amount of API remaining in the depot. After filtration step through a 0.45 µm PTFE filter for the meloxicam solution and through a 0.20 µm hydrophilic filter for the tamsulosin solution, the solutions were analyzed by UPLC using the methods described in the Supplementary Materials (Tables S1 and S2).

### 2.3. Quantification of DMSO in the Release Medium

The solvent exchange process, which finally leads to the formation of the depot upon injection in an aqueous environment, was followed using a similar setup and procedure to those used for in vitro release tests, in order to investigate the effects of the formulation parameters on the DMSO release.

DMSO was quantified by RP–HPLC using the method detailed in the Supplementary Materials (Table S3).

### 2.4. In Vivo Studies

Six male Wistar rats of ca. 250–350 g each were subcutaneously injected in the interscapular area with 160 µL of F01 and F02 formulations containing meloxicam. Up to 28 days, at predetermined time points, 250 µL of blood was withdrawn from the jugular vein and transferred into tubes containing K_2_EDTA. After collection, the samples were centrifuged in order to recover the plasma, which was then stored in polypropylene tubes at −80 °C until analysis. The in vivo phase was performed by Avogadro LS (Toulouse, France). Animal housing and care comply with the recommendations of Directive 2010/63/EU. The animal facilities at Avogadro LS have the authorization number D 31 188 01 obtained on November 23, 2017 from the French Veterinary Authorities and the animal care and use program is AAALAC accredited. The study plan was favorably assessed by the Avogadro LS Animal Ethics Committee.

Quantification of meloxicam in plasma was performed by Eurofins ADME Bioanalyses (Vergèze, France).

After euthanasia, the injection sites were excised from all animals and stored in labeled vials at −80 °C until analysis. Explant treatment and analysis were performed by MedinCell (Montpellier, France).

### 2.5. Characterization of the Depots from the In Vivo Study

#### 2.5.1. X-ray Microtomography

X-ray microtomography was used to analyze the internal structure of the polymeric depots obtained from the in vivo study without altering it. This analysis uses X-rays to create cross sections of a depot that were used to recreate a virtual model. The analyses were performed at the Faculté des Sciences de Montpellier within the MRI-CRBM laboratory, using an EasyTom 150kV from RX Solutions (Chavanod, France). The samples were placed in specific tubes with a diameter of 1.5 cm and analyzed without any prior treatment with the following key scanning parameters:

- Source voltage: 40 kV;
- Frame rate: 4.5 F/s;
- Digital gain: 3;
- Intensity: 200 µA;
- Final resolution (voxel size): 9 µm;
- Focal spot: small < 20 µm.

Xact software was used to reconstruct cross-section images from the cone–beam X-ray projections with the following parameters:

- Ring artifact correction: 5;
- Custom contrasts: from 0 to 4.

#### 2.5.2. Quantification of API

Retrieved explants were placed in Falcon tubes and cut in several pieces to allow full dissolution, and the required amount of acetonitrile was added. The falcon tubes were then closed and left under stirring on a roller mixer at room temperature until complete dissolution of the explants.

When dissolutions were complete, the tubes were centrifuged for 5 min at 3500 rpm, and an aliquot of the supernatant (1.0 mL) was withdrawn and transferred into a UPLC glass vial for meloxicam quantification by RP–UPLC following the method summarized in the Supplementary Materials (Table S1).

#### 2.5.3. Gel Permeation Chromatography

GPC is a common analytical tool used to characterize polymers by determining their molecular weights (Mp, Mw, Mn) and dispersity (Đ). As the technique relies on the separation by the size of the polymer chains, recording the GPC chromatogram of a specific composition at the starting point of a study allows for the detection of any alteration of the molecular weight distribution over time, thus monitoring the polymer integrity at each time point.

GPC analyses were performed on treated depots obtained from the in vivo study using a Waters Alliance e2695 separation module with a Waters 2414 refractive index (RI) detector. The instrument was equipped with one Waters Styragel 4.6 mm × 30 mm guard column and a series of 7.8 mm × 30 mm Waters Styragel separation columns (HR 4, HR 3, and HR 2) kept at 35 °C. The samples were run in tetrahydrofuran (THF) stabilized with butylated hydroxytoluene (BHT) at a concentration of 10 mg/mL and at a flow rate of 1.0 mL/min. The system was calibrated with narrow poly(styrene) standards (193 kDa–370 Da, Waters Polystyrene Standards Kit WAT011588 from Waters (Milford, MA, USA).

During explant treatment, the depots were initially dissolved in acetonitrile. Then, the solvent was evaporated using a SpeedVac centrifugal concentrator (MiVAC Duo concentrator) from Genevac (Ipswich, United Kingdom) to obtain dried polymers and allow for GPC analyses. Approximately 30 mg of the treated depot was weighed in an empty 3 mL labeled glass vial. Exact masses were recorded. The required THF volume was added to reach a final polymer concentration of around 10 mg/mL. The vial was then closed and left overnight to stir at room temperature until the complete dissolution of the copolymers.

When dissolution was complete, around 1.5 mL of solution was withdrawn from the glass vial and filtered through a 0.45 µm PTFE filter into an HPLC glass vial.

Analyses were performed in triplicates; molecular weight values were rounded to the closest hundred and typical errors were <5%. Data were obtained using the Empower GPC data analysis software from Waters (Milford, MA, USA). Mn, Mw, and Đ were recorded 28 days after formulation injection and compared to initial values.

## 3. Results

**Figure 2:**
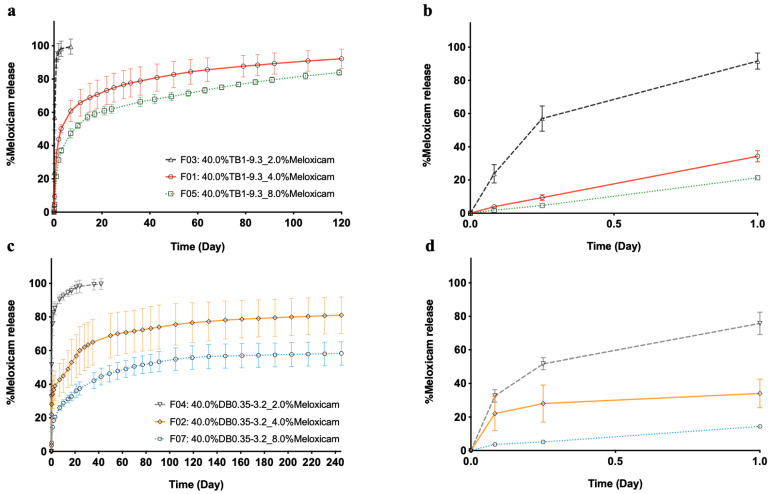
Meloxicam delivery from PEG–PTMC triblock (**a**) and diblock (**c**) copolymer-based formulations with different API:copolymer ratios and the respective zoomed released over the first 24 h (**b**,**d**).

Figure 2 shows the in vitro release of meloxicam from PEG–PTMC triblock (TB1-9.3) and diblock (DB0.35–3.2) based formulations with different API:copolymer ratios. It must be noted that, while the 2% (wt.%) meloxicam formulations were clear solutions, the 4 and 8% (wt.%) meloxicam formulations were suspensions. A more sustained release is obtained in both TB- and DB-based formulations with meloxicam in suspension, with delivery durations beyond 120 days. On the contrary, meloxicam is released quickly from depots resulting from solution formulations, irrespective of the type of copolymer used.

**Figure 3:**
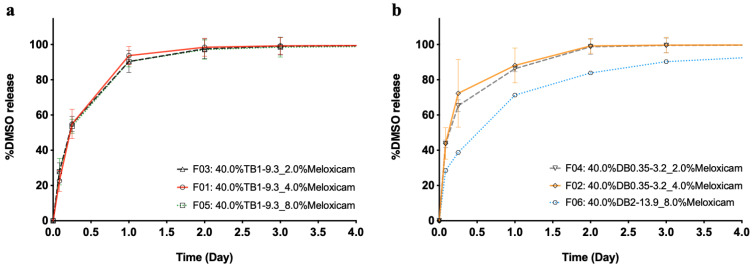
DMSO release from (m)PEG–PTMC triblock (**a**) and diblock (**b**) copolymer-based formulations with different API:copolymer ratios.

Figure 3 displays the in vitro release of DMSO from (m)PEG–PTMC DB- and TB-based formulations upon injection into PBS. The phase exchange is slightly quicker in TB-based formulations, with ca. 95% of the DMSO diffused after 1 day of immersion into the release medium, compared to 85% in the case of the DB containing injectables. In both cases, all DMSO had been released from the depot 2 days after the initiation of the study. It can be noted that the solvent release kinetics is similar, regardless of the API content within the formulations, for both copolymer types.

**Figure 4:**
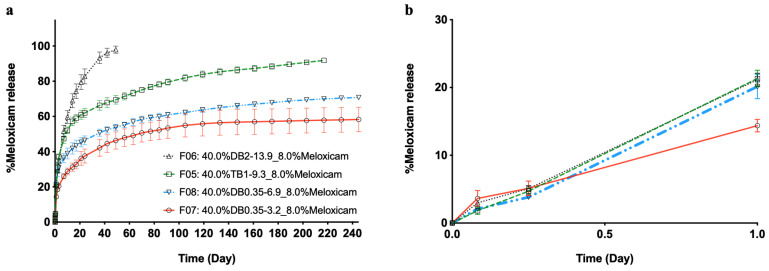
Meloxicam release from (m)PEG–PTMC suspension formulations with different triblock and diblock copolymers (**a**) and the zoomed released over first 24 h (**b**).

The in vitro release of meloxicam from depots resulting from suspension formulations (containing 8% (wt.%) API), i.e., with particles of the drug dispersed in the solution of the polymer, is shown in Figure 4. Formulations were prepared with the same concentration of either TB or DB of (m)PEG–PTMC copolymers with different (m)PEG and PTMC molecular weights. It can be observed that meloxicam release spans go from ca. 40 days to over 240 days depending on the copolymer composition.

**Figure 5:**
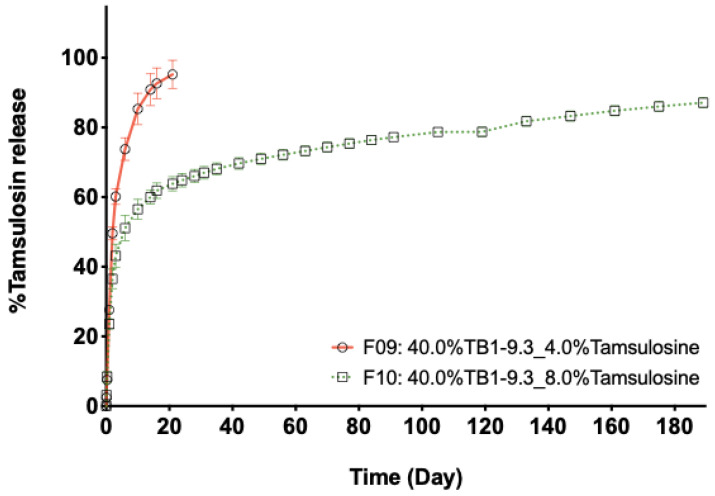
Tamsulosin release from PEG–PTMC solution (F09) and suspension (F10) formulations with the same type and content of triblock PEG–PTMC (TB1-9.3_40.0%).

Figure 5 shows the in vitro release of tamsulosin from depots of formulations prepared with the same TB PEG–PTMC and similar polymer content but different drug amounts: 4 and 8% (wt.%), which resulted in solution (F09) and suspension (F10) injectables, respectively. It can be observed that the release duration from F09 based depots is shorter, with a complete release after ca. 20 days of immersion in the release medium. The release from F10 based depots is characterized by an initial burst, followed by a sustained delivery up to at least 180 days, when the experiment was stopped.

**Figure 6:**
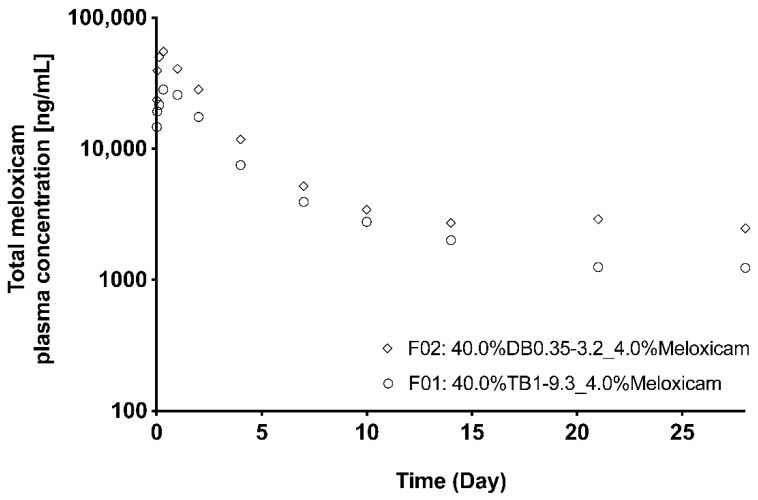
Meloxicam plasmatic concentration in rats following the administration of an (m)PEG–PTMC-based suspension formulation with either a triblock or a diblock copolymer.

Figure 6 shows the plasmatic concentration of meloxicam in rats after the subcutaneous administration of (m)PEG–PTMC based formulations prepared with either one DB or one TB copolymer. The evolution of the plasmatic concentration is similar for both formulations, with a higher initial meloxicam peak followed by a sustained delivery until the end of the study, at 28 days. The amount of nonreleased meloxicam in the recovered explants was 47% for F01 and 23% for F02 prepared with TB1–9.3 and DB0.35–3.2, respectively, suggesting that the TB copolymer allowed for more controlled delivery of the encapsulated drug. This is confirmed by the lower C_max_ and AUC obtained when administering the TB-based formulation (Table 3). It must be noted that the amount of (m)PEG–PTMC (40 wt.%) and the ratio of meloxicam:copolymer (1:10 wt:wt) was the same for both formulations.

**Figure 7:**
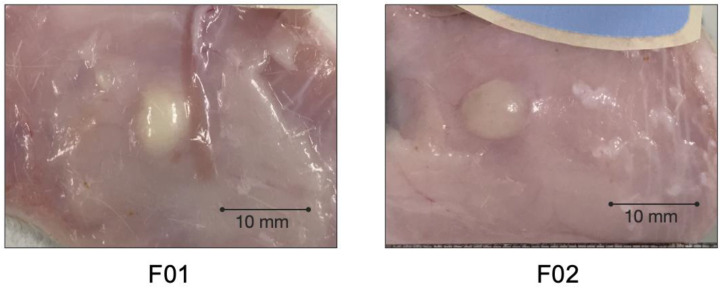
Photos of the injection sites 28 days post administration to rats of a PEG–PTMC-based suspension formulations with either a triblock (F01) or a diblock (F02) copolymer.

Photos of the injection sites at the end of the pharmacokinetic study are displayed in Figure 7. The polymeric depot can be clearly distinguished from the subcutaneous environment. No evident local tolerance reactions could be observed macroscopically. The (m)PEG–PTMC depots were recovered as discrete, solid but soft bodies upon excision. Isolation from the surrounding tissue was performed easily since the depots were only covered by a thin layer of tissue.

**Figure 8:**
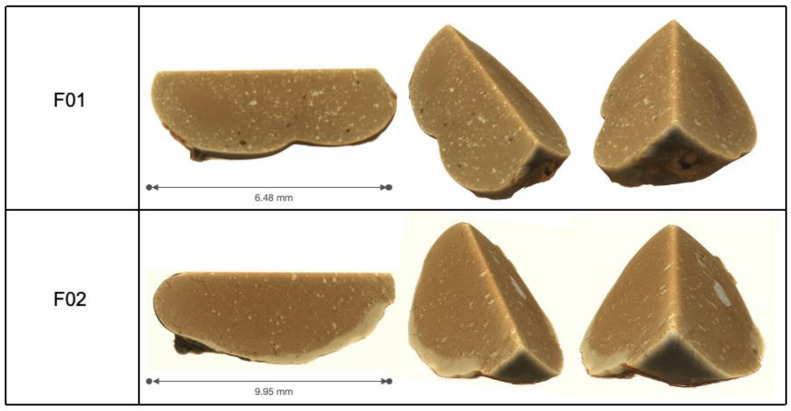
Three-dimensional X-ray microtomography reconstructions of PEG–PTMC triblock (F01) or diblock (F02) depots explanted 28 days post administration to rats.

Figure 8 shows representative X-ray microtomography three-dimensional reconstructions of (m)PEG–PTMC based matrices excised from the rats at the end of the pharmacokinetic study. Depots appear nonporous and homogenous. Several particles of different contrast, indicating a different density of the material, can be distinguished uniformly distributed in both depots.

**Figure 9:**
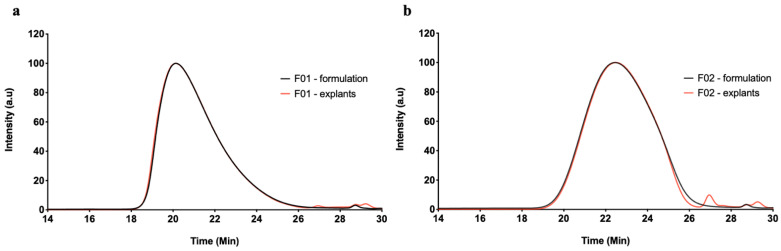
GPC–RI chromatograms of F01 (**a**) and F02 (**b**) (m)PEG–PTMC based formulations (black) and depots explanted 28 days post administration to rats (red).

Figure 9 displays characteristic GPC–RI chromatograms of (m)PEG–PTMC based formulations and depots explanted from the subcutaneous environment of rats 28 days post administration. The GPC profile of the formulation prior to administration (in black) is superposable to that of the depots four weeks after injection to the rats (in red), except for some low molecular weight species eluting after 26 min. The latter species are likely to correspond to biological species that were extracted together with the polymers prior to analysis, as previously observed in our laboratories for other ISFD in vivo tests (data not shown). Consequently, the polymers present similar M_n_, M_w_, M_p_ and Ð, summarized in Table 4, pre- and post-in vivo administration.

## 4. Discussion

Polycarbonate-based polymers are widely accepted in the biomedical field because of their well-known biocompatibility and bioresorbability [17,23,24]. Based on these characteristics and their physical properties, these polymers are being used for the fabrication of sutures and have been broadly explored as materials for the manufacture of scaffolds for tissue regeneration [26,27]. Variants of these polymers such as PEG–polycarbonates block copolymers have been also studied as excipients for the formulation of controlled drug delivery pharmaceutical forms such as micelles, nanoparticles, or thermogelling systems with very promising results [28,29,30,31]. On top of it, previous studies in our laboratories have demonstrated that certain compositions of (m)PEG–PTMC are soluble in biocompatible organic solvents and that, albeit slow, these copolymers undergo degradation and bioresorption in vivo. Therefore, the present study aimed to evaluate the utilization of (m)PEG–PTMC diblock and triblock copolymers for the formulation of long-acting injectables based on solvent exchange mechanism. These tests were conceived since solvent-exchange-based ISFD technologies offer a wide range of tunable parameters to achieve diverse release profiles and kinetics. Hence, some of the studies described in this manuscript were designed to assess whether the utilization of different ratios of hydrophilic PEG and hydrophobic PTMC within (m)PEG:PTMC could be used as a way to tune the drug release kinetics, similarly to other amphiphilic-based controlled delivery systems [20].

In vitro drug delivery tests confirmed that (m)PEG–PTMC based formulations could be used as in situ forming depot systems based on a solvent exchange mechanism. These formulations were based on the dissolution of (m)PEG–PTMC copolymers in DMSO in which a drug (meloxicam or tamsulosin) was subsequently added to form either solutions or homogeneous suspensions within the polymeric vehicle. Once injected into an aqueous environment, the DMSO diffused into the surrounding medium and the copolymers, which were designed to be insoluble in water, precipitated to form a polymeric depot that entrapped the drug within. Interestingly, it was observed that the solvent diffused very rapidly to the surrounding medium and that, additionally, the kinetics of release of DMSO were similar independently of the polymeric composition. These observations differ from the behavior noticed in ISFD LAI formulated with PEG-polyesters, where the solvent exchange timespan could be tuned with the utilization of copolymers with different PEG:polyester ratios [20]. This quick solvent exchange on (m)PEG–PTMC based injectables may be explained by the high hydrophobicity of the aliphatic polycarbonate chains, which forces the diffusion of the polar DMSO to the surrounding aqueous medium [32].

A drawback of the quick diffusion of the DMSO from the forming polymeric depots is that a high fraction of the solubilized drug may leave the depot together with the solvent before the full precipitation of the polymeric matrix [33]. This phenomenon explains the almost immediate release of the drug when this technology was tested in full solution formulations (2% meloxicam and 4% tamsulosin). However, long release durations were obtained when formulating with suspended drugs. Furthermore, it was demonstrated that the initial burst could be decreased by increasing the quantity of the suspended drug in the injectables. The fraction of the drug that is being released from the depots formed with suspension formulations depends on the amount of solubilized API; formulations with the more suspended drug have a lower proportion of the cargo released in the early time points during the phase exchange. Based on these observations, it is reasonable to suggest that, for long-term release durations, (m)PEG–PTMC ISFD LAI would be most suited for suspensions with a low soluble fraction. Therefore, the potential of these copolymers for the formulation of this type of product was evaluated with meloxicam as a model drug as suspension.

The ratio between hydrophilic and hydrophobic chains has been identified in the past as a key parameter to modulate the release kinetics from different pharmaceutical forms formulated with amphiphilic copolymers. Similarly, our results demonstrate that meloxicam delivery kinetics could be tuned by formulating with (m)PEG–PTMC copolymers with different (m)PEG and PTMC molecular weights. Release duration spanned from ca. 40 to more than 240 days in vitro from formulations with the same polymer content and drug:(m)PEG–PTMC ratio but with different diblock and triblock copolymers. These results may be explained by the different hygroscopy of the resulting depots as a function of the hydrophilic:hydrophobic ratio of the polymeric chains. This may influence the water influx into the matrices and, in turn, change the diffusion kinetics of the encapsulated drug into the aqueous environment.

A pharmacokinetic study in rats confirmed that the controlled release observed during the in vitro tests was also maintained when administering (m)PEG–PTMC based injectables in vivo. Indeed, both triblock- and diblock-based suspension formulations achieved a sustained release of meloxicam for 28 days. It can likely be anticipated that the release would have lasted significantly longer since a high amount of nonreleased drug was recovered from the explants at the end of the study. The release profiles were comparable to those typically obtained with other solvent-exchange technologies, with a higher initial concentration due to a quick meloxicam delivery during the phase-exchange phase, followed by a sustained release upon depot formation. Similar to what is observed in the in vitro tests, the in vivo release kinetics, and therefore, the pharmacokinetic parameters could be tuned with the utilization of different (m)PEG–PTMC copolymers as functional excipients. The enhanced control of the triblock-based formulations (F01) might be due to the higher molecular weight of these copolymers when compared to the diblock copolymers used for formulating F02. Additionally, this divergence might be explained by the different hydrophilicity of the copolymers based on their PEG:PTMC molecular weight ratio, which may impact the hygroscopicity of the resulting depots.

On top of the promising release profiles, results from this in vivo study suggest good biocompatibility of (m)PEG–PTMC based depots in the subcutaneous environment; no evident local tolerance issues could be observed macroscopically at the injection site during neither the in vivo phase (no swelling, no erythema) nor in the subcutis at the end of the study. Furthermore, the polymeric meloxicam reservoirs were covered by a very thin tissue layer, indicating a well-tolerated foreign body reaction following the administration of these injectables. Further dedicated studies, including histopathology, should be carried out for evaluating further and confirming these promising results.

The X-Ray tomography analysis of the explants from the injection sites at the end of the pharmacokinetic study revealed that the interior of the depots was a homogenous polymeric matrix with low porosity. This characterization also allowed to observe particles of different density spread through the depot; it can be hypothesized that these could be the nonreleased meloxicam, which was entrapped upon precipitation of the suspension formulation. The apparent very low porosity of these depots could be at least partially explained by the very quick DMSO diffusion upon administration of the formulations. Previous studies with ISFD have demonstrated that rapid phase exchanges yield depots with very small pores [33,34]. It is logical to postulate that this low porosity also results in a limited swelling of the depots in an aqueous environment. Indeed, excessive swelling has been identified as one of the potential drawbacks of polyester-based ISFDs, due to the accumulation of liquid within the polymeric matrix [35].

Lastly, the GPC analysis of the explants displayed that 28 days after the administration in vivo, the copolymers constituting the depots had an almost identical chromatographic profile to that of the pristine (m)PEG–PTMC in the formulations prior to injection. The absence of smaller polymeric entities within the explants upon in vivo administration is in line with previous studies that have analyzed the degradation of polycarbonate-based technologies. It is well documented that polycarbonate-based materials undergo surface erosion by enzymatic degradation, which results in the loss of mass of the matrix without altering the overall polymer molecular weight distribution within. Furthermore, it has been demonstrated that the kinetics of degradation is dependent on the size of the copolymer chains; the larger the polycarbonate chains forming the material, the quicker the degradation [9]. Based on these studies, it can be hypothesized that the (m)PEG–PTMC depots evaluated in this study are being slowly degraded on their surface because of the relatively low molecular weight of the polycarbonate chains of the copolymers. This gradual but slow erosion of the material may be allowing the diffusion of the meloxicam molecules once available within the surrounding fluids, which may explain the long-term release kinetics obtained during the pharmacokinetic study.

Taken together, the results shown in this article demonstrate the potential of (m)PEG–PTMC copolymers as versatile excipients for the formulation of in situ forming depot long-acting injectable formulations by solvent exchange. The good biocompatibility and depot integrity observed upon a 28-days exploratory pharmacokinetic study in rats suggest that this technology is best suited for the formulation of suspension injectables aiming for very long-term drug delivery. Outcomes from this study are particularly promising for applications where the very long-term delivery of an anti-inflammatory drug such as meloxicam may be desirable.

## 5. Conclusions

This article describes, for the first time, the utilization of (m)PEG–PTMC diblock and triblock copolymers for the formulation of in situ forming depot-based long-acting injectables, allowing a durable sustained delivery of a therapeutic molecule.

Drug release durations of up to several months can be achieved in vitro with suspension formulations using these copolymers. The delivery kinetics can be tuned by changing several parameters such as the length of the PEG or PTMC within the copolymers or the ratio between the drug and API.

A pharmacokinetic study in rats confirmed the sustained delivery of meloxicam from a PEG–PTMC based injectable formulation and further demonstrated a good in vivo tolerability for this technology.

## Figures and Tables

**Table 1:** Compositions of formulations delivering meloxicam.

Formulation	Triblock TBm-n	Diblock DBs-t	Total Polymer Content (wt.%)	API Content (wt.%)	DMSO Content (wt.%)
F01	1–9.3	-	40.0	4.0	56.0
F02	-	0.35–3.2	40.0	4.0	56.0
F03	1–9.3	-	40.0	2.0	58.0
F04	-	0.35–3.2	40.0	2.0	58.0
F05	1–9.3	-	40.0	8.0	52.0
F06	-	2–13.9	40.0	8.0	52.0
F07	-	0.35–3.2	40.0	8.0	52.0
F08	-	0.35–6.9	40.0	8.0	52.0

**Table 2:** Compositions of formulations delivering tamsulosin.

Formulation	Triblock TBm-n	Diblock DBs-t	Total Polymer Content (wt.%)	API Content (wt.%)	DMSO Content (wt.%)
F09	1–9.3	-	40.0	4.0	56.0
F10	1–9.3	-	40.0	8.0	52.0

**Table 3:** PK parameters obtained from F01 and F02 formulations.

Formulation	PK Parameter	Mean	SD	Median
F01	C_max__D (kg × g/mL/mg)	1084	286	1087
	T_max_ (h)	-	-	8 (8–24) *
	AUC_last__D (h × kg × ng/mL/mg)	114,192	20,661	120,437
F02	C_max__D (kg × ng/mL/mg)	1979	305	2041
	T_max_ (h)	-	-	8 (3–8) *
	AUC_last__D (h × kg × ng/mL/mg)	174,597	25,340	175,775

* Median (min–max).

**Table 4:** GPC–RI values obtained from F01 and F02 formulations pre- and post-in vivo administration.

Formulation	Test item	M_n_ (kDa)	M_w_ (kDa)	M_p_ (kDa)	Ð
F01	Formulation—t0	13.9	19.8	24.4	1.4
	Explants—28 days	14.3	20.7	25.6	1.5
F02	Formulation—t0	7.3	10.3	10.0	1.4
	Explants—28 days	7.5	10.4	9.8	1.4

## Data Availability

The data presented in this study are available on request from the corresponding author.

## Supplementary Materials

The following are available online at https://www.mdpi.com/article/10.3390/pharmaceutics13050605/s1, Table S1: UPLC method for the quantification of meloxicam, Table S2: UPLC method for the quantification of tamsulosin, Table S3. HPLC method for the quantification of DMSO.
