# Supplementary material for: Poly(ethylene glycol)-b-poly(1,3-trimethylene carbonate) Copolymers for the Formulation of In Situ Forming Depot Long-Acting Injectables
Source: Pharmaceutics. 2021 Apr 22;13(5):605. doi: 10.3390/pharmaceutics13050605 (PMC8146374; doi:10.3390/pharmaceutics13050605)
Supplement: Supplementary file 1 [file pharmaceutics-13-00605-s001.zip › pharmaceutics-1153325-SI.pdf]

pharmaceutics

Supplementary Materials: Poly(ethylene glycol)-*b*-poly(1,3-trimethylene carbonate) Copolymers for the Formulation of In Situ Forming Depot Long-Acting Injectables

Marie-Emérentienne Cagnon, Silvio Curia, Juliette Serindoux, Jean-Manuel Cros, Feifei Ng, and Adolfo Lopez-Noriega

Table S1. UPLC method for the quantification of meloxicam.

**Column**	**Acquity UPLC BEH C18 column (1.7 µm, 130 Å, 2.1x50 mm)**		
Pre-column	Acquity Col.In-Line fiters 0.50 mm		
Column temperature	+30 °C		
Mobile phase A	Acetonitrile + 0.1% TFA		
Mobile phase B	H_2O + 0.1% TFA		
Volume injected	1 µL		
Flow rate	0.3 mL/min		
Retention time	2.0 min		
Wavelength	352 nm		
	**Time (min)**	**%A**	**%B**
Gradient	**0**	40	60
	**2.70**	50	50
	**2.85**	100	0
	**3.35**	100	0
	**3.85**	40	60
	**7**	40	60

Table S2. UPLC method for the quantification of tamsulosin**.**

**Column**	**Acquity UPLC BEH C8 column (1.7 µm, 130 Å, 2.1x50 mm)**		
Pre-column	Acquity Col.In-Line fiters 0.50 mm		
Column temperature	+30 °C		
Mobile phase A	Acetonitrile + 0.1% Formic acid		
Mobile phase B	H_2O + 0.1% Formic acid		
Volume injected	1 µL		
Flow rate	0.7 mL/min		
Retention time	1.25 min		
Wavelength	225 nm		
Gradient	**Time (min)**	**%A**	**%B**
	0	95	5
	2	5	95
	3	5	95
	3.5	95	5
	5	95	5

Table S3. HPLC method for the quantification of DMSO.

**Column**	**Kinetox HPLC EVO C18 column (5 µm, 100 Å, 150x4.6 mm)**			
Security guard	ULTRA Cartridges UHPLC C18 for 4.6mm ID columns			
Column temperature	+25°C			
Mobile phase A	Acetonitrile			
Mobile phase B	H_2O			
Volume injected	10 µL			
Retention time	2.2 min			
Wavelength	210 nm			
Gradient	**Time (min)**	**Flow rate (mL/min)**	**%A**	**%B**
	**0**	0.8	1	99
	**2**	0.8	1	99
	**4.5**	1.0	80	20
	**6.5**	1.0	80	20
	**9**	1.0	1	99
	**10**	0.8	1	99
	**13**	0.8	1	99
